# How is the quality of randomized controlled trials (RCTs) for acupuncture treatment of post-stroke aphasia? A report quality assessment

**DOI:** 10.1371/journal.pone.0308704

**Published:** 2024-10-25

**Authors:** Chenyang Qin, Shizhe Deng, Boxuan Li, Weiming Zhu, Chaoda Liu, Hailun Jiang, Bifang Zhuo, Menglong Zhang, Yuanhao Lyu, Junjie Chen, Shihao Chi, Beidi Cao, Xinming Yang, Zhihong Meng

**Affiliations:** National Clinical Research Center for Chinese Medicine Acupuncture and Moxibustion, First Teaching Hospital of Tianjin University of Traditional Chinese Medicine, Tianjin, China; The Second Affiliated Hospital of Shandong First Medical University, CHINA

## Abstract

**Objective:**

This study aimed to assess the quality of randomized controlled trials (RCTs) that have reported the use of acupuncture for the treatment of post-stroke aphasia (PSA).

**Methods:**

We systematically searched PubMed, Embase, Cochrane Library, Web of Science, Chinese National Knowledge Infrastructure (CNKI), Wanfang data Information Site, and China Science and Technology Journal Database from January 2013 to June 2023. RCTs utilizing acupuncture as an intervention for the treatment of post-stroke aphasia were included in this study. The overall quality score (OQS) of RCTs was independently evaluated by two researchers using the Consolidated Standards for Reporting Trials (CONSORT) and the Standards for Reporting Interventions in Controlled Trials of Acupuncture (STRICTA) guidelines, with the agreement between researchers calculated using Cohen’s kappa statistics.

**Results:**

In conclusion, we included 38 RCTs in this study. The median OQS of the 38 RCTs was 13 (minimum 8, maximum 20) based on the CONSORT statement. Out of all CONSORT items, 10 (27%) had a positive rate of greater than 80%, while 17 (46%) had a positive rate of less than 10%. The median OQS of the 38 RCTs was 12 (minimum 6, maximum 14) based on the STRICTA guideline. Within the STRICTA guideline, 6 items (35%) had a positive rate of greater than 80%, and 3 items (18%) had a positive rate of less than 10%. Most items based on the CONSORT and STRICTA guidelines were observed to have a perfect or good degree of agreement.

**Conclusions:**

The overall reporting quality of RCTs for acupuncture treatment of PSA was found to be suboptimal. Notably, the reporting quality of the STRICTA guideline is higher compared to the CONSORT statement. Therefore, strict adherence to both the CONSORT and STRICTA statements is recommended to enhance the quality of RCT reports on acupuncture treatment for post-stroke aphasia.

## Introduction

Stroke has emerged as a critical global health issue, ranking as the second leading cause of death and the foremost cause of disability worldwide [1, 2]. Generally speaking, a variety of functional impairments following stroke will occur, like motor and sensory disorders, language deficits, dysphagia, and so on [3]. Approximately one third of stroke survivors experience aphasia [4], which severely affects patients’ social interactions, mental health and quality of life. Aphasia, characterized by a collection of acquired receptive and expressive language impairments [5], is one of the most devastating clinical entities after stroke, resulting in a disorder in spontaneous speech, comprehending spoken words, reading, writing, and others [6]. In addition, post-stroke aphasia (PSA) patients were equally related to higher costs of stroke care. A study has demonstrated that PSA patients increased hospitalization costs by $971.35 and length of stay by 0.66 days as compared to non-aphasia patients [7]. Similarly, another study has shown that the rehabilitation costs of PSA patients were about $1,700 higher than that of non-aphasia patients [8]. This undoubtedly severely aggravated the difficulty of clinical rehabilitation and the economic burden of medical and health care. Therefore, there is an urgent need for an effective and easily applicable clinical intervention to treat PSA, aiming to enhance the quality of life for PSA patients and alleviate the societal healthcare burden.

A review of clinical trials on PSA found that interventions for PSA include stimulation therapy, speech and language therapy (SLT), pharmacological therapy and others [9]. Nevertheless, there is inadequate evidence to support the long-term efficacy caused by stimulation therapy for PSA [10]; receiving face-to-face SLT with a therapist can be burdensome for patients, as many PSA patients may face severe communication barriers [11]; there is no high-level evidence that pharmacological therapy gains a significant improvement in language for PSA [12].

Acupuncture originates from Traditional Chinese Medicine, which is widely used against a variety of diseases [13]. Similarly, due to its safety and efficacy, acupuncture is widely used as a complementary and alternative therapy for the rehabilitation of post-stroke aphasia in China. In recent years, the number of clinical trials of acupuncture for PSA has increased. A meta-analysis of randomized controlled trials reported that acupuncture is effective on improvement in language function of PSA [14]. Randomized controlled trials (RCTs) have been viewed as the gold standard of clinical trial design, and it is considered the highest level of evidence in a clinical study [15]. However, incomplete and inadequate RCTs represent a waste that reduces the usefulness of research and failed to benefit clinical practice [16] and it might mislead clinical interventions, ultimately affecting therapeutic effects. As a consequence, it is valuable to evaluate the quality of RCTs of acupuncture for PSA.

The Consolidated Standards for Reporting Trials (CONSORT) [17, 18], established in 1996 and updated in 2010, aims at improving the transparency and rigor of RCTs. The Standards for Reporting Interventions in Controlled Trials of Acupuncture (STRICTA) [19, 20], an extension of the CONSORT statement, was published in 2001 and revised in 2010 to help researchers fully report details of clinical studies on acupuncture. As far as we know, there is no study assessing the quality of acupuncture for PSA using CONSORT and STRICTA statements. Consequently, the main goal of this study is to evaluate the reporting quality of RCTs of acupuncture for PSA based on the above two guidelines. This is intended to acquire preliminary data that can aid in advancing clinical trials on acupuncture treatment for PSA and identifying potential issues currently existing in clinical trials, thereby providing a reference for future clinical research.

## Materials and methods

### Search strategy

**Fig 1:**
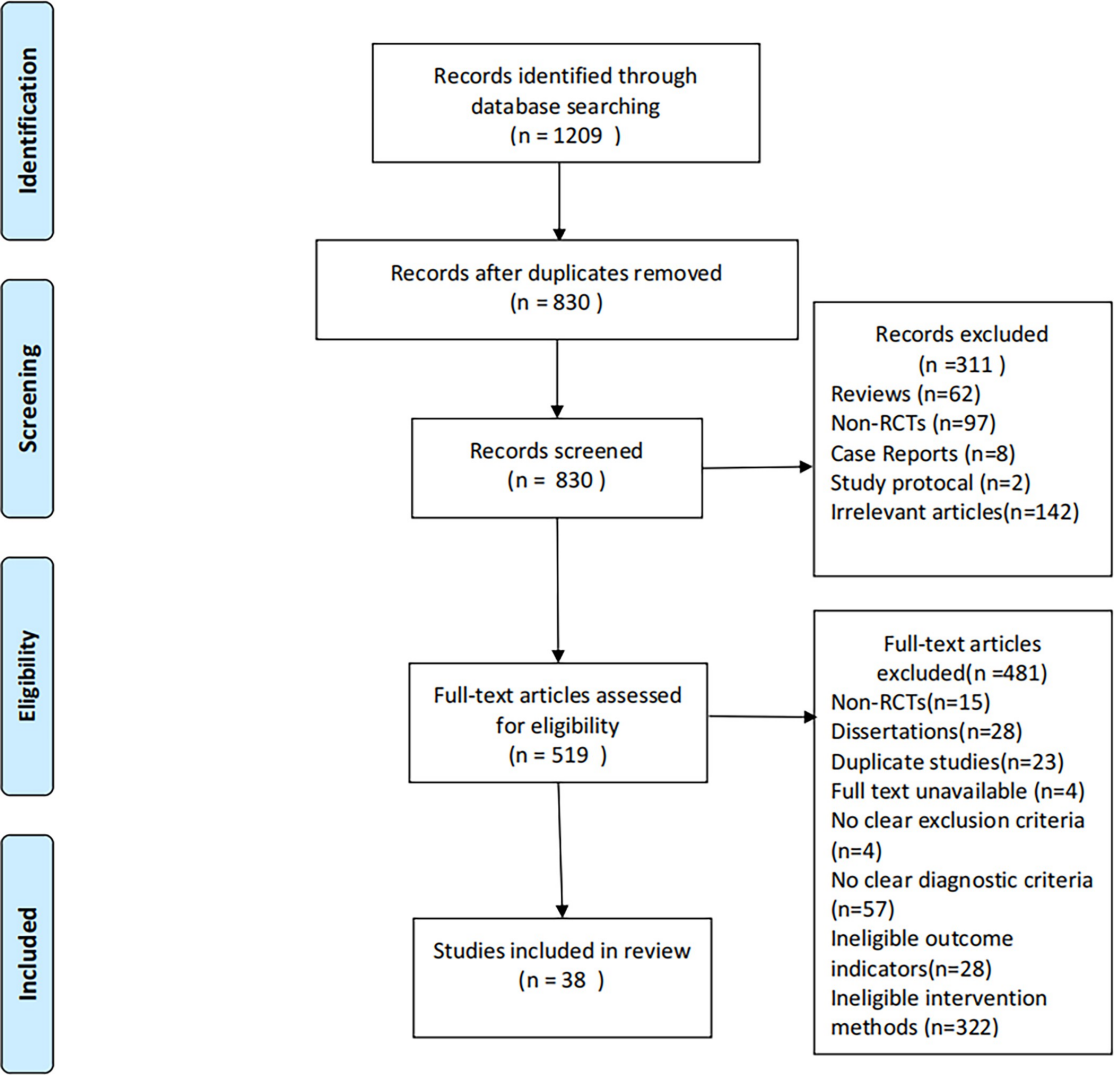
Flow chart of selection process. The entire selection process resulted in a total of 38 studies being included in the analysis.

The following online databases were searched from January 2013 to June 2023: PubMed, Embase, Cochrane Library, Web of Science, China National Knowledge Infrastructure (CNKI), China Science and Technology Journal Database and Wanfang data Information Site. The main combinations of search terms used in Chinese and English were as follows: (acupuncture OR scalp acupuncture OR tongue acupuncture OR body acupuncture OR manual acupuncture OR acupuncture therapy OR electroacupuncture OR needle OR acupoint) AND (aphasia) AND (stroke OR apoplexy OR cerebral apoplexy OR cerebral infarction OR cerebral hemorrhage) AND (RCT OR randomized OR randomised OR randomized trial OR randomized controlled trials OR RCTs). The details of the search process are displayed in Fig 1.

### Included and excluded criteria

#### Types of studies

We included RCTs in our assessment to examine the efficacy of acupuncture interventions for PSA. The study excluded reports that met the following criteria: (1) abstract only or full text not available; or (2) non-randomized, reviews, cross-over RCTs, case reports, retrospective studies, conference papers, dissertations, case-control studies and duplicate publications.

#### Types of participants

We included subjects who have been clearly diagnosed as PSA by referring to clearly diagnostic criteria, regardless of gender, ethnicity, occupation, and other demographic factors.

Aphasia caused by other diseases is excluded.

#### Types of intervention

Different methods of acupuncture for example scalp acupuncture, tongue acupuncture, electroacupuncture and acupoint-sites, were all included.

Included RCTs met the following criteria: (1) in the experimental group acupuncture was implemented alone, and a placebo or conventional treatment or sham acupuncture was used in the control group; or (2) when the intervention in the experimental group was acupuncture combined with other therapies, the intervention in the control group must be same as other therapies of the experimental group.

Excluded RCTs met the following criteria: (1) studies in which the experimental group of patients was served by acupuncture combined with medication; or (2) studies that compared the effectiveness of different acupuncture therapies; or (3) studies with a total effective rate alone as an outcome indicator.

### Study selection and data extraction

First, the researcher (C Y Q) used NoteExpress 3.8.0 software to eliminate duplicate studies. Next, two researchers (C Y Q and B X L) independently screened the titles and abstracts of the studies based on inclusion and exclusion criteria. After the initial screening, the researchers reviewed the full texts of all potentially eligible studies to make the final selection. Any reports with contentious eligibility were labeled as ’uncertain,’ and a senior researcher (Z H M) made the ultimate decision regarding their inclusion.

Subsequently, two researchers (C Y Q and B X L) utilized Microsoft Excel 2019 16.0 software to extract general information from the included studies. This information encompassed details such as the author, publication year, number of subjects, intervention methods, treatment duration, and other elements relevant to the assessment based on CONSORT and STRICTA guidelines.

### Assessment of reporting quality

Two researchers (C Y Q and B X L) individually assessed the reporting quality of included RCTs based on the CONSORT and STRICTA statements. To evaluate the degree of agreement between the two researchers, Cohen’s κ-statistic was calculated using IBM SPSS Statistics 23.0 software. Agreement was judged as perfect if κ was > 0.8, good if 0.6 < κ ≤ 0.8, moderate if 0.4 < κ ≤ 0.6, fair if 0.2 < κ ≤ 0.4 and poor if κ was ≤ 0.2.

Each of the Thirty-seven items included in the CONSORT 2010 was scored to calculate an overall quality score (OQS). Each of seventeen items in the STRICTA statements was also scored. For scoring the quality of items, one point was given if the item-related information was mentioned in the study, and zero points if it was not mentioned or was unclear (S1 Table).

## Results

### Search results

Initially, a total of 1209 relevant reports were identified from seven databases. 830 reports remained after the exclusion of duplicates. After reading the titles, abstracts and full text, 38 RCTs were ultimately extracted for further assessment. Fig 1 outlines the whole selection process for this study, and the general characteristics of the 38 included RCTs are indicated in Table 1.

**Table 1:** General characteristics of included 38 studies.

Included Studies	Publication Year	Sample	Intervention	Course of Treatment
Zhang J W [21]	2014	120	Acupuncture VS Conventional therapy	30d
He J F [22]	2015	80	Acupuncture+SLT VS SLT	8w
Wang N [23]	2015	40	Scalp Acupuncture+SLT VS SLT	4w
Xiong J [24]	2016	64	Xingnao Kaiqiao Acupuncture+SLT VS SLT	5w
Tong X N [25]	2017	84	Acupuncture+SLT VS SLT	4w
Liu Y [26]	2017	98	Xingnao Kaiqiao Acupuncture+SLT VS SLT	5w
Xing J [27]	2017	70	Tongdu Kaiqiao Acupuncture+SLT VS SLT	30d
Yang L [28]	2017	90	Electroacupuncture+SLT VS SLT	2w
Teng Y Y [29]	2017	91	Scalp Acupuncture+SLT VS SLT	30d
Chen X F [30]	2018	40	Scalp Acupuncture+SLT VS SLT	4w
Zheng Y B [31]	2018	120	Electroacupuncture+SLT VS SLT	4w
Wang Q [32]	2018	71	Tongue Acupuncture+SLT VS SLT	6w
Yu Q P [33]	2018	60	Acupuncture+SLT VS SLT	4w
Weng P X [34]	2019	90	Acupuncture+SLT VS SLT	30d
Xiao Y K [35]	2019	100	Scalp Acupuncture+SLT VS SLT	30d
Yin X J [36]	2019	48	Acupuncture+SLT VS SLT	4w
Li Z G [37]	2019	80	Tongue Acupuncture+SLT VS SLT	90d
Ye J S [38]	2019	80	Scalp Acupuncture+rTMS+SLT VS rTMS+SLT	3w
Lin M [39]	2019	80	Electroacupuncture+SLT VS SLT	2w
Liu G [40]	2019	68	Xingnao Kaiyin Acupuncture+SLT VS SLT	4w
Yuan X L [41]	2020	68	Acupuncture+Hyperbaric Oxygen +SLT VS Hyperbaric Oxygen+SLT	4w
Qiu L F [42]	2020	60	Acupuncture+SLT VS SLT	8w
Wang Y [43]	2020	100	Electroacupuncture+SLT VS SLT	30d
Fan Q Y [44]	2020	82	Acupuncture+SLT VS SLT	90d
Ma Y P [45]	2020	40	Electroacupuncture+SLT VS SLT	3w
Liu W [46]	2020	60	Scalp Acupuncture+SLT VS SLT	8w
Zhang R M [47]	2020	76	Scalp Acupuncture+SLT VS SLT	8w
Ren C Z [48]	2020	70	Jingou Diaoyu Acupuncture+SLT VS SLT	4w
Wang C H [49]	2021	89	Acupuncture+SLT VS SLT	4w
He J L [50]	2021	112	Xingnao Kaiyin Acupuncture+SLT VS SLT	8w
Wang L P [51]	2021	86	Tongue Acupuncture+ aNMES +SLT VS aNMES +SLT	20d
Wang S Q [52]	2021	80	Electroacupuncture+ brTMS VS brTMS	4w
Jia Z Y [53]	2021	102	Tongyang Kaiqiao Acupuncture+SLT VS SLT	4w
Wei J H [54]	2021	86	Acupuncture+SLT VS SLT	8w
Chen X Y [55]	2021	82	Xingnao Kaiyin Acupuncture+SLT VS SLT	8w
Wang Y L [56]	2021	66	Scalp Acupuncture+SLT VS SLT	8w
Wu L L [57]	2022	82	Acupuncture+SLT VS SLT	8w
Sun J B [58]	2023	80	Kaiqiao Xingshen Acupuncture+SLT+ ctDCS VS SLT+ctDCS	6w

^a^NMES: Neuromuscular Electrical Stimulation

^b^rTMS: repetitive Transcranial Magnetic Stimulation

^c^tDCS: transcranial Direct Current Stimulation

### Characteristics of included trials

**Fig 2:**
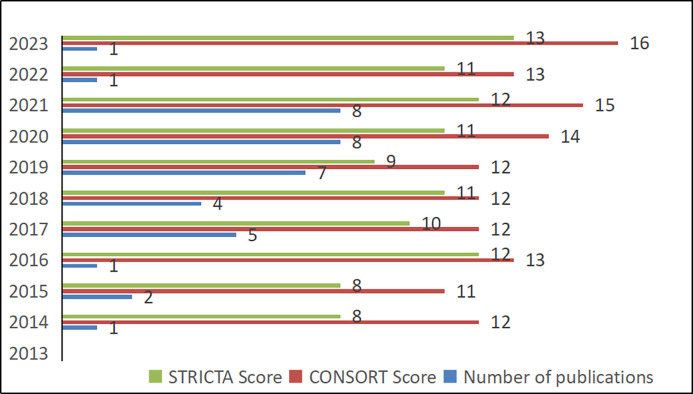
Information of included RCTs. The blue bars is the number of publications. The red and green bars represent the average scores of OQS based on CONSORT and STRICTA guidelines respectively in each year.

**Fig 3:**
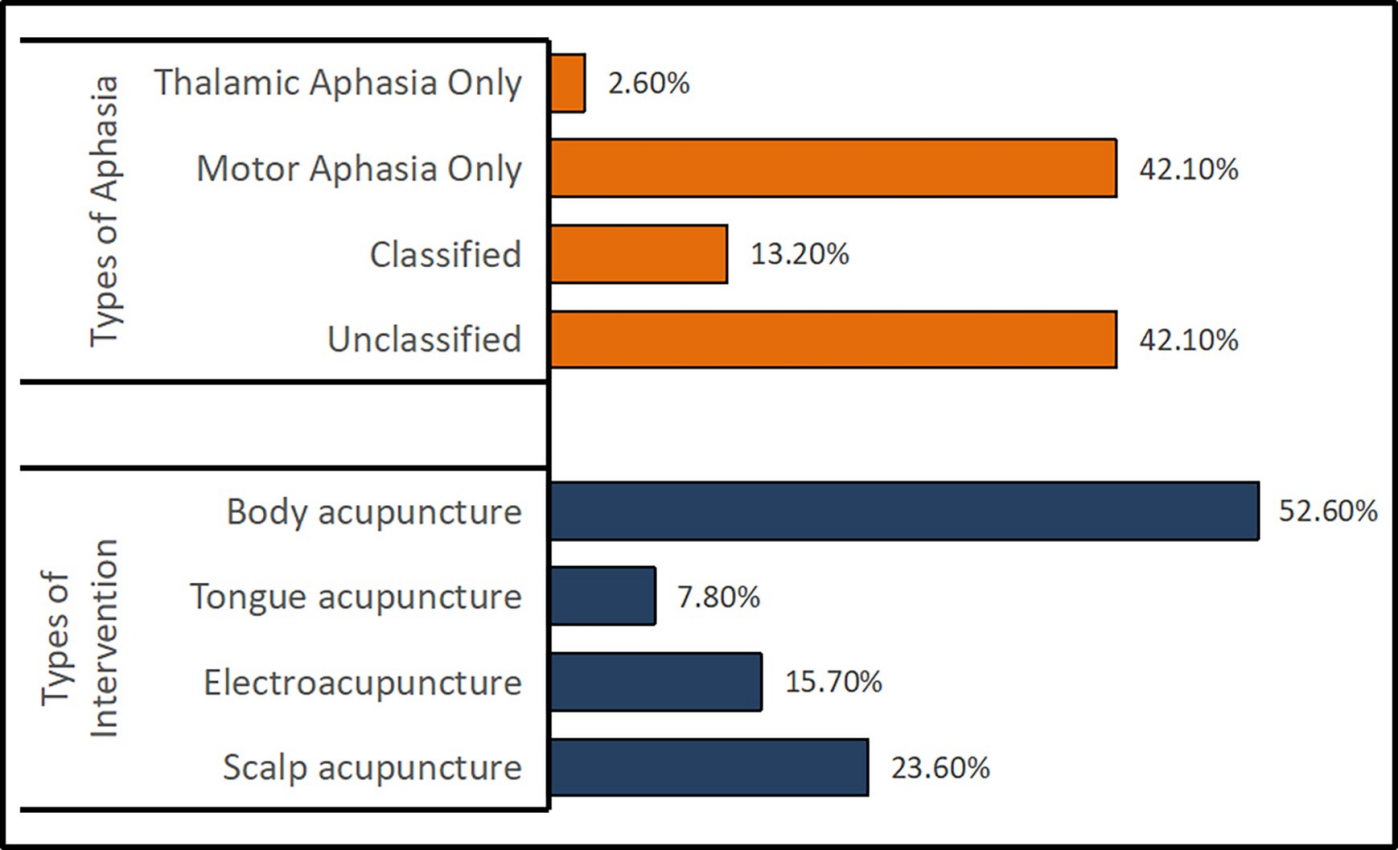
Information of included RCTs. The orange bars represent the types of aphasia, and the blue bars represent the types of interventions.

Out of the 38 studies, 13 (34.2%) were published between 2013 and 2018, while 25 (65.8%) were published from 2019 to the present (see Fig 2). All 38 RCTs were conducted by Chinese researchers. The sample size ranged from 40 to 120 (Median: 80). Only one report (2.6%) used acupuncture alone in the experimental group, while 37 reports (97.3%) utilized acupuncture in combination with other therapies. The acupuncture treatment course varied from 2 weeks to 12 weeks (Median: 4 weeks). Only 9 studies (23.7%) reported their source of funding. The main interventions in the treatment group included scalp acupuncture (23.6%), electroacupuncture (15.7%), tongue acupuncture (7.8%), and body acupuncture (52.6%). There were 5 studies (13.2%) that classified aphasia, while 16 studies (42.1%) did not report the type of aphasia. Additionally, 16 studies (42.1%) included patients with motor aphasia, and 1 study (2.6%) involved patients with thalamic aphasia (see Fig 3 and Table 2).

**Table 2:** The details of OQS assessed with CONSORT statement (n = 38).

Items	Items No.	Description	NO.of positive trials	%	Cohen’s κ coefficient	95%CI
Title and abstract						
	1.a	Identification as a randomized trial in the title.	0	0	1	1
	1.b	Structured summary of trial design, method, results, and conclusions (for specific guidance see CONSORT for abstracts).	36	95	0.787	0.38 to 1.00
Introduction						
Background and objectives	2.a	Scientific background and explanation of rationale.	33	87	0.874	0.63 to 1.00
	2.b	Specific objectives or hypotheses.	18	47	0.735	0.52 to 0.95
Methods						
Trial design	3.a	Description of trial design (such as parallel, factorial) including allocation ratio.	0	0	1	1
	3.b	Important changes made to the method after the trial commencement (such as eligibility criteria), with reason.	0	0	1	1
Participants	4.a	Eligibility criteria for participants.	38	100	1	1
	4.b	Settings and locations where the data were collected.	20	53	0.840	0.67 to 1.0
Interventions	5	Sufficient details of each intervention, including how and when they were administered to allow replication.	32	84	0.525	0.19 to 0.86
Outcomes	6.a	Defined pre-specified primary and secondary outcome measures, including how and when they were assessed.	19	50	0.732	0.51 to 0.95
	6.b	Any changes to trial outcomes after the beginning of the trial, and the reasons.	0	0	1	1
Sample size	7.a	How was the sample size determined.	0	0	1	1
	7.b	Explanation of any interim analyses and stopping guidelines when applicable.	0	0	1	1
Randomization						
Sequence generation	8.a	Methods used to generate the random allocation sequence.	33	87	0.874	0.63 to 1.0
	8.b	Type of randomization; details of any restrictions (such as blocking and block size).	1	3	1	1
Allocation concealment	9	The mechanism used to implement the random allocation sequence (such as sequentially numbered containers), description of any steps taken to conceal the sequence until the assignment of interventions.	1	3	1	1
Implementation	10	Who had generated the random allocation sequence, who had enrolled participants, and who had assigned participants to the interventions.	0	0	1	1
Blinding	11.a	Who had been blinded (for example, participants, care providers, those assessing outcomes) and how.	0	0	1	1
	11.b	Description of the similarity of interventions if relevant.	32	84	0.469	0.10 to 0.86
Statistical methods	12.a	Statistical methods used to compare the primary and secondary outcomes in each group.	34	89	0.642	0.19 to 1.0
	12.b	Methods for additional analyses, such as subgroup analyses and adjusted analyses.	0	0	1	1
Results						
Participant flow	13.a	For each group, the numbers of randomly assigned participants, whether they received the intended treatment, and whether the primary outcome was analyzed.	22	58	0.890	0.74 to 1.0
	13.b	Losses and exclusions after randomization in each group and the corresponding reasons.	4	11	0.874	0.63 to 1.0
Recruitment	14.a	Dates defining the periods of recruitment and follow-ups.	36	95	1	1
	14.b	The reason why the trial was stopped or terminated.	0	0	1	1
Baseline data	15	A table showing baseline demographics and clinical characteristics for each group.	9	24	1	1
Numbers analyzed	16	For each group, the number of participants (denominator) included in each analysis and whether the analysis had been performed by the originally assigned groups.	19	50	0.894	0.75 to 1.0
Outcomes and estimation	17.a	The primary and secondary outcomes result for each group, the estimated effect size and its precision (such as 95% confidence interval).	38	100	1	1
	17.b	The presentation of both absolute and relative effect sizes was recommended for binary outcomes.	0	0	1	1
Ancillary analyses	18	Results of any other performed analysis, including subgroup analysis and adjusted analysis, distinguishing the pre-specified from the exploratory.	0	0	1	1
Harms	19	All important adverse or unintended effects in each group (for specific guidelines see CONSORT for adverse effects)	2	5	0.787	0.38 to 1.0
Discussion						
Limitations	20	Trial limitations, addressing sources of potential bias, imprecision, and, if relevant, multiplicity of analyses.	14	37	0.785	0.59 to 0.98
Generalizability	21	Generalizability (external validity, applicability) of the trial findings	17	45	0.790	0.60 to 0.98
Interpretation	22	Interpretation consistent with results, balancing benefits and side effects, and considering other relevant evidence.	34	89	0.737	0.52 to 0.95
Registration	23	Registration number and name of trial registry.	0	0	1	1
Protocol	24	Where the full trial protocol can be accessed, if available	0	0	1	1
Funding	25	Sources of funding or other supports (such as drug supply), role of funders	9	24	1	1

### Quality of the reports

#### Reporting quality score based on CONSORT items

Table 2 presents the data regarding the overall quality of reporting based on the CONSORT statement. Among the 38 RCTs, the median OQS was 13, ranging from a minimum of 8 to a maximum of 20. Items such as "structured summary," "scientific background," "eligibility criteria," "interventions," "random allocation sequence," "description of the similarity of interventions," "statistical methods," "recruitment and follow-ups," "outcomes result," and "interpretation" were reported in a sufficiently detailed manner, with positive rates exceeding 80%. These items accounted for 27% of all the checklist items.

Conversely, items related to "title," "changes to methods," "trial design," "changes to outcomes," "sample size," "interim analyses," "type of randomization," "allocation concealment," "implementation," "blinding," "additional analyses," "reasoning for stopping," "absolute and relative effect sizes," "ancillary analyses," "harms," "registration," and "study protocol" were reported less thoroughly, with a positive rate of less than 10%. These items accounted for 46% of all checklist items.

The degree of agreement among the items was generally good or perfect, except for items (Interventions) and 11b (Description of the similarity of interventions), which exhibited a moderate degree of agreement.

#### Reporting quality score based on STRICTA items

Table 3 displays the data regarding the overall quality of reporting based on the STRICTA statement. Among the 38 RCTs, the median OQS was 12, with a minimum score of 6 and a maximum of 14. Items such as "style of acupuncture," "names of points," "needle stimulation," "needle retention time," "number of treatment sessions," and "frequency and duration of treatment" were adequately reported, with a positive rate exceeding 80%. These items constituted 35% of all checklist items.

**Table 3:** The details of OQS assessed with STRICTA checklist (n = 38).

Items	Description	NO.of positive trials	%	Cohen’s κ coefficient	95%CI
1. Acupuncture rationale (explanations and examples)	1a) Style of acupuncture (e.g., Traditional Chinese Medicine, Japanese, Korean, Western medical, Five Element, ear acupuncture).	38	100	1	1
	1b) Reasoning for provided treatment, based on historical context, literature sources, or consensus methods, with references where appropriate.	27	71	0.594	0.30 to 0.88
	1c) Extent to which treatment varied.	9	24	0.583	0.31 to 0.86
2. Details of needling (explanations and examples)	2a) Number of needle insertions per subject per session (mean and range where relevant).	2	5	1	1
	2b) Names (or location if there was no standard name) of points used (uni/bilateral).	38	100	1	1
	2c) Depth of insertion, based on a specified unit of measurement, or on a particular tissue level.	27	71	0.822	0.63 to 1.0
	2d) Response sought (e.g.de qi or muscle twitch response).	25	66	0.879	0.71 to 1.0
	2e) Needle stimulation (e.g. manual, electrical).	38	100	1	1
	2f) Needle retention time.	35	92	1	1
	2g) Needle type (diameter, length, and manufacturer or material).	16	42	0.841	0.67 to 1.0
3. Treatment regimen (Explanations and examples)	3a) Number of treatment sessions.	36	95	0.787	0.38 to 1.0
	3b) Frequency and duration of treatment sessions	34	89	0.843	0.54 to 1.0
4. Other components of treatment (explanations and examples)	4a) Details of other interventions administered to the acupuncture group (e.g. moxibustion, cupping, herbs, exercises, lifestyle change).	28	74	0.565	0.26 to 0.87
	4b) Setting and context of treatment, including instructions to practitioners, information and explanations to patients.	1	3	1	1
5. Practitioner background (explanations and examples)	5) Description of participating acupuncturists (qualifications or professional affiliations, years in acupuncture practice, other relevant experiences).	2	5	1	1
6. Control or comparator interventions (explanations and examples)	6a) The rationale for the control or comparator in the context of the research question, with sources that justify this choice.	25	66	0.744	0.51 to 0.98
	6b) A precise description of the control or comparator. If sham acupuncture or any other type of acupuncture-like control was used, provide details as in items 1–3 above.	28	74	0.564	0.28 to 0.85

On the other hand, items related to the "number of needle insertions," "setting and context of treatment," and "description of participating acupuncturists" had a positive rate below 10%, accounting for 18% of all items. Most RCTs provided sufficient information regarding the treatment regimen and needling details. Items 1b (reasoning for provided treatment), 1c (Extent to which treatment varied), 4a (details of other interventions), and 6b (a precise description of the control or comparator) demonstrated a moderate degree of agreement, while the remaining items exhibited a good or perfect degree of agreement.

## Discussion

In this study, we conducted a systematic search of RCTs on acupuncture for the treatment of PSA published in the last decade. We evaluated the quality of the included reports using the CONSORT and STRICTA guidelines. Unfortunately, the results of reporting quality were found to be unsatisfactory. The overall average reporting rate was 35.6% based on CONSORT and 63.3% based on STRICTA statements, which is consistent with the findings of previous reports [59, 60].

The low reporting quality based on the CONSORT statement was a cause for concern. The median overall quality score (OQS) of the 38 RCTs was 13, which is less than 35% of the total points. Most items of the CONSORT statement were inadequately reported or overlooked, with significant deficiencies observed in the trial methods section, particularly in items related to trial design, sample size, randomization, and blinding.

Notably, none of the included reports indicated in their titles that they were RCTs, which may complicate the identification of RCTs. Additionally, none of the studies explicitly described their trial design, such as randomized, parallel, or factorial design. Clearly defining the trial design can reduce potential misunderstandings in research data and enhance the transparency of the trials [61]. Inappropriate trial design not only leads to considerable resource waste but also affects the reliability of the clinical evidence it generates [62]. According to a study published in *Lancet*, about 85% of funding is wasted each year due to improper trial design and other reasons [63].

None of the studies reported the item of sample size. Sample size calculation details are required to be reported so that the reader can critically judge whether the sample size calculation was realistic and reproduces it [64]. An inadequate sample size may fail to detect clinical effects, produce confounding factors, and biases. Adequate sample size calculation ensures that financial and medical resources are used efficiently to achieve reliable clinical outcomes based on clinically significant differences [65, 66].

Randomization and allocation concealment are key methodologic parts of RCTs. Only one study (3%) reported sequence generation and allocation concealment, despite 87% of the included studies mentioning the randomization method used. The reporting rate of the methodologic items has been low and has not been improved [67, 68]. Moreover, no one study reported who had generated the random allocation sequence. As one of the key methodological parts of RCTs, the implementation of allocation concealment is conducive to reduce selection bias, enhance baseline comparability, and minimize trial result heterogeneity [69]. Likewise, the biases of statistical outcome will occur in trials that use inadequate allocation concealment methods [70]. Thus, it is crucial to pay more attention to randomization and select a good allocation concealment method to improve report quality and prevent various biases.

Furthermore, none of the studies provided details of blinding. Blinding is comprised of single-blinded, double-blinded, and triple-blinded [71]. Blinding can be implemented in a wide range of distinct procedures of trials and correct blinding is vital for improving the validity of outcome assessment and preventing potential biases [72, 73]. Hróbjartsson et al [74] observed that the treatment effect was overestimated in unblinded RCTs. Compared with pharmacologic trials, blinding patients and intervenors simultaneously is more difficult to carry out in trials on acupuncture as its particularity. At present, blinding methods in acupuncture trials consist of nonpenetration acupuncture, shallow puncture, non-acupoints puncture and so on [75–77]. Despite it being difficult in blinding in acupuncture trials, researchers should make every effort to implement blinding methods to improve the scientific rigor and validity of their trials.

Surprisingly, none of the RCTs reported the accessibility of their study protocols and registration numbers. Study protocols serve as the foundation for conducting clinical trials, describing the trial’s purpose, design, methods, and statistical information. Registration of trials and the availability of protocols ensure that clinical trial information is publicly accessible, aligning with the principles of evidence-based medicine [78]. Trial registration and protocols also protect the informed rights and interests of study participants, enhance public trust in clinical trials, and enable tracking of trial outcomes and quality control of trial processes and results [79].

Regarding the quality assessment based on the STRICTA guideline, it was rated as moderate. The median OQS of the 38 RCTs was 12, which was greater than 70% of the total points. While most items of the STRICTA guidelines were reported in more than 70% of trials, indicating relatively complete descriptions of acupuncture interventions, some items, such as "number of needles," "setting and context of treatment," and "qualifications of acupuncturists," were not fully addressed. Only 2 RCTs (5%) reported the number of needle insertions and the qualifications of acupuncturists, and 1 RCT (3%) reported the treatment setting. Notably, a study has shown that the number of needles and sessions are related to the treatment outcomes [80]. Besides, more than half of the RCTs did not report the type of acupuncture, and some only mentioned the length of acupuncture without information like diameter and manufacturer. Neglect of the explanation of acupuncture details not only affects the internal validity of the clinical results but also influences other researchers to replicate the intervention [81]. On top of that, different acupuncturists can make a difference impact on studies because of the characteristics of acupuncture, so the lack of report on the qualification of acupuncturists may affect the generalizability of trial results [82]. Therefore, it is crucial to provide clear descriptions of acupuncture interventions in compliance with STRICTA guidelines.

Generally, there are several common issues observed in the reporting of acupuncture RCTs. A study evaluating the reporting quality of acupuncture RCTs has revealed that the reporting quality of Chinese-language acupuncture RCTs needs significant improvement when compared to English-language acupuncture RCTs. Additionally, the reporting rates of certain items, such as trial methods, differ by over 50% between Chinese and English RCTs [83]. This may be closely linked to the lack of emphasis among Chinese researchers on clinical trial registration, resulting in selective reporting. At the same time, we must acknowledge that positive research outcomes are more likely to be published compared to negative results. Therefore, giving greater importance to clinical trial registration can significantly reduce the likelihood of publication bias. In an assessment of the quality of RCTs for acupuncture treatment of female urinary incontinence, it was noted that funding support can influence CONSORT scores, with funded RCTs more likely to receive higher scores [84]. This can partly explain that funding providers and project supervisors play a crucial role throughout the entire RCT trial. The importance of trial protocols is emphasized in the quality assessment of RCTs for acupuncture treatment of low back pain, as these protocols provide an overview and detailed information about the entire study [68]. The evaluation of the quality of RCTs for acupuncture treatment of acute herpes zoster highlights that a lack of proper sample size estimation can lead to a decrease in test performance, affecting the authenticity and reliability of study results [60]. These challenges mirror the observations made in the evaluation presented in this study.

There are a few limitations to our study. First and foremost, the number of included RCTs was relatively small. We excluded literature that compared different acupuncture methods and studies in which intervention types were inconsistent between the experimental and control groups, which could potentially impact our research results. However, this reduction in heterogeneity among studies was achieved. A more comprehensive assessment would require the inclusion of a larger number of studies for quality evaluation. Second, it is important to note that all the included RCTs were conducted in China. This might be attributed to the fact that the mechanism of acupuncture for PSA is not entirely clear, even though it has been proven effective in treating aphasia. Consequently, there are relatively few relevant international clinical trials. Lastly, we only included RCTs published in the past 10 years. It remains uncertain whether the inclusion of RCTs published before 2013 would alter the composition of our reports or the outcomes. However, recent studies are more likely to identify potential issues in ongoing RCTs, making them relatively more clinically instructive.

## Conclusions

In summary, this study has provided an overview of the reporting characteristics of RCTs on acupuncture for PSA and has highlighted that the overall quality of these reports is suboptimal. It is worth noting that the quality of reports based on the CONSORT statement is relatively lower, while those following the STRICTA checklist tend to be of better quality. RCTs with suboptimal quality may compromise their scientific validity and their ability to provide effective evidence for clinical treatment. Therefore, we recommend that researchers should rigorously adhere to both the CONSORT and STRICTA statements and focus on enhancing the quality of methodological aspects such as randomization, allocation concealment, blinding, and so on in their future reports.

## Supporting information

S1 Checklist PRISMA 2009 checklist. (DOC)

S1 Table Data extraction. (XLSX)
